# PReS-FINAL-2039: Evaluation of local activity of wrists and finger joints damage in patients with juvenile idiopathic arthritis: the values of radiology methods

**DOI:** 10.1186/1546-0096-11-S2-P52

**Published:** 2013-12-05

**Authors:** D Alekseev, I Nikishina, A Smirnov, O Pushkova, A Volkov

**Affiliations:** 1 Pediatric Rheumatology, Federal State Budgetary Institution, Scientific Research Institute for Rheumatology, RAMS, Moscow, Russian Federation; 2 Radiology, Federal State Budgetary Institution, Scientific Research Institute for Rheumatology, RAMS, Moscow, Russian Federation; 3 Functional Diagnostics, Federal State Budgetary Institution, Scientific Research Institute for Rheumatology, RAMS, Moscow, Russian Federation

## Introduction

Juvenile idiopathic arthritis is a heterogeneous group of chronic inflammatory diseases with high risk of progressive articular damage. Clinical examination is the basis for assessing of the affected joints. Modern radiological techniques provide additional important information about the status of the damaged joints, allow the identification of subclinical inflammation.

## Objectives

To investigate the additional capabilities of assessing local activity of finger joints of the hands and wrists in patients with different types of JIA by the modern methods of radiology diagnostic of articular pathology (magnetic resonance imaging (MRI) and ultrasonography (US)) in comparison with clinical research.

## Methods

20 patients with JIA (systemic, n = 3; polyarticular, n = 11; oligoarticular, n = 6) were studied. Mean age was 12,3 years (range 6,5-17,1). Mean disease duration was 21,2 months (range 8-61). On clinical evaluation recorded the tenderness/pain on motion, swelling, limitation of movement. MRI (ESAOTE O-Scan) and US (ESAOTE MyLab; linear probe, 6-18 MHz) assessment of fingers and wrists joints was performed with the main focus of research on the signs of local activity: MRI - the presence of fluid in the joint cavity, US - the presence of joint effusion, synovial hyperplasia, power Doppler signal. We compared the results of clinical examination and radiology diagnostics.

## Results

On clinical examination, synovitis was detected in 103 finger joints of the hands, in 9 wrist joints. On MRI and/or US synovitis was identified in 145 of finger joints of the hands, 13 wrist joints. Thus, by radiological methods subclinical activity was detected in 42 (28.9%) of finger joints of the hands, 4 (30.7%) - of wrists. Of them, 7 joints of the fingers and 1 of the wrists revealed a significant degree of activity on MRI and/or US, including a pronounced increase of vascularization of the synovial membrane (by means of power Doppler).

In 20 finger joints clinically indicated only the presence of pain. Of them, in 6 joints revealed clear signs of activity on US: in 4 joints - small or moderate severity; in 2 joints - significant changes (moderate amount of joint effusion, substantial vascularization of the synovial membrane).

In 2 patients the signs of synovitis associated with manifestations of dactylitis.

## Conclusion

In the studied group of patients with different subtypes of JIA, the use of MRI and ultrasound possible to detect subclinical synovitis in most patients. The use of modern radiological methods in patients with JIA can verify the exact local activity, improves detection of subclinical synovitis.

## Disclosure of interest

None declared.

